# New Determinants of Aminoglycoside Resistance and Their Association with the Class 1 Integron Gene Cassettes in *Trueperella pyogenes*

**DOI:** 10.3390/ijms21124230

**Published:** 2020-06-13

**Authors:** Ewelina Kwiecień, Ilona Stefańska, Dorota Chrobak-Chmiel, Agnieszka Sałamaszyńska-Guz, Magdalena Rzewuska

**Affiliations:** Department of Preclinical Sciences, Institute of Veterinary Medicine, Warsaw University of Life Sciences, Ciszewskiego 8 St., 02-786 Warsaw, Poland; ewelina1708@gmail.com (E.K.); i.stefanska@gmail.com (I.S.); dorota.chrobak@wp.pl (D.C.-C.); asalam@tlen.pl (A.S.-G.)

**Keywords:** aminoglycosides, antimicrobial resistance, gene cassette, integron, pathogen, *Trueperella pyogenes*

## Abstract

*Trueperella pyogenes* is an important opportunistic animal pathogen. Different antimicrobials, including aminoglycosides, are used to treat *T. pyogenes* infections. The aim of the present study was to evaluate aminoglycoside susceptibility and to detect aminoglycoside resistance determinants in 86 *T. pyogenes* isolates of different origin. Minimum inhibitory concentration of gentamicin, streptomycin, and kanamycin was determined using a standard broth microdilution method. Genetic elements associated with aminoglycoside resistance were investigated by PCR and DNA sequencing. All studied isolates were susceptible to gentamicin, but 32.6% and 11.6% of them were classified as resistant to streptomycin and kanamycin, respectively. A total of 30 (34.9%) isolates contained class 1 integrons. Class 1 integron gene cassettes carrying aminoglycoside resistance genes, *aadA11* and *aadA9*, were found in seven and two isolates, respectively. Additionally, the *aadA9* gene found in six isolates was not associated with mobile genetic elements. Moreover, other, not carried by gene cassettes, aminoglycoside resistance genes, *strA*-*strB* and *aph(3’)-IIIa*, were also detected. Most importantly, this is the first description of all reported genes in *T. pyogenes*. Nevertheless, the relevance of the resistance phenotype to genotype was not perfectly matched in 14 isolates. Therefore, further investigations are needed to fully explain aminoglycoside resistance mechanisms in *T. pyogenes*.

## 1. Introduction

*Trueperella pyogenes* (formerly *Arcanobacterium pyogenes*) is a Gram-positive, nonmotile, nonspore-forming, irregular rod. This bacterium is a common inhabitant of mucous membranes of the upper respiratory, gastrointestinal, and urogenital tracts of animals [1,2,3]. However, *T. pyogenes* can be pathogenic for both domestic and wild animals and causes different diseases, including mastitis, metritis, balanoposthitis, pneumonia, and abscesses in various organs and tissues [1,3,4,5]. Those infections are especially important in swine, cattle, and small ruminants, since they lead to serious economic losses [4,5,6,7,8]. In humans *T. pyogenes* infections are rare, but may occur especially in immunocompromised individuals and those who have contact with infected animals [9,10].

Aminoglycosides, β-lactam antibiotics, tetracyclines, macrolides, and fluoroquinolones are antimicrobials commonly used to treat infections caused by different Actinomycetales, including *T. pyogenes*. However, extensive antibiotic use may be the reason for increasing drug resistance in *T. pyogenes* [4,5,6,7,11]. Although *T. pyogenes* has been known for a long time, its mechanisms of antimicrobial resistance are still poorly understood. It was previously reported that different mobile genetic elements may play an important role in the acquisition of antimicrobial resistance in this bacterium. The *tetW* gene, which determines the tetracycline resistance in *T. pyogenes,* is located on transposons [12]. Two genes associated with the macrolide resistance, *ermB* and *ermX*, are carried by variable genetic elements in *T. pyogenes*, such as plasmids or transposons [13,14]. Additionally, it was found that in the *T. pyogenes* resistance to trimethoprim, chloramphenicol, aminoglycosides, and β-lactam antibiotics, integrons may also play an essential role [5,7,15].

Integrons are genetic elements that are composed of three main structural components, including the integrase gene (*intI*), the recombination site (*attI*), and two promoters (P_c_ and P_int_) [16]. Until now, several classes of integrons have been recognized based on a class-specific amino acid sequence of the integrase [16]. Integrons are able to capture and express antimicrobial resistance genes located in mobile gene cassettes. Gene cassettes’ integration and excision occur by site-specific recombination between the *attI* site of an integron and the *attC* site of a gene cassette. Integrons containing gene cassettes may be located in chromosomes or in mobile genetic elements, such as plasmids and transposons. Therefore, integrons play an important role in distribution of antimicrobial resistance genes among bacteria often belonging to different genera and species [17,18,19,20,21]. The role of integrons in the spread of antimicrobial resistance genes is especially well described in Gram-negative bacteria [22]. Some Gram-positive bacteria may also be reservoirs of integrons, including *Staphylococcus* spp. [23], *Enterococcus* spp. [24], *Corynebacterium* spp. [25], *Streptococcus* spp. [26], and *T. pyogenes* [5,7,15]. However, issues concerning transfer and dissemination of integrons between *T. pyogenes* and other bacteria, Gram-positive and Gram-negative, are still unclear and require further investigation.

The enzymatic modification is a common mechanism of the aminoglycoside resistance in various bacteria. There are three main types of aminoglycoside-modifying enzymes: *N*-acetyltransferases (ACCs), *O*-nucleotidyltransferases (ANTs), and *O*-phosphotransferases (ADHs). Genes encoding those enzymes in various bacteria are usually related to mobile genetic elements such as plasmids or transposons [27]. However, to date, little is known about mechanisms of the aminoglycoside resistance in *T. pyogenes*.

The aim of this study was to determine a minimum inhibitory concentration (MIC) of selected aminoglycosides and to detect aminoglycoside resistance determinants in 86 *T. pyogenes* isolates of different origin, including unique isolates from European bison (*Bison bonasus*). In our study various sources of tested isolates allowed us to compare the aminoglycoside resistance among livestock (cattle, swine, goats, sheep) and wild animal (European bison, antelope) *T. pyogenes* isolates.

## 2. Results

### 2.1. Aminoglycoside Susceptibility

Susceptibility to three aminoglycosides was tested for 86 *T. pyogenes* isolates from various animal species. MICs of tested aminoglycosides for all *T. pyogenes* isolates are presented in Table S1 of Supplementary Materials. Distribution of MIC values of gentamicin, streptomycin, and kanamycin are shown in Table 1. The susceptibility testing results showed that all isolates were susceptible to gentamicin (100%). However, 28 (32.6%) and 10 (11.6%) isolates were resistant to streptomycin and kanamycin, respectively. The MIC_50_ and MIC_90_ (concentrations that inhibited growth of 50% and 90% of isolates, respectively) values of tested antibiotics are presented in Table 1. The prevalence of aminoglycoside resistance, regarding the origin of *T. pyogenes* isolates, is shown in Table 2. Generally, bovine and swine isolates were resistant to tested aminoglycosides, except gentamycin, at a higher percentage than other isolates. The isolates from small ruminants (2/13) were resistant only to streptomycin. Interestingly, two *T. pyogenes* isolates from European bison were resistant to kanamycin, while the majority of them were susceptible to all tested aminoglycosides. Moreover, European bison isolates were characterized by lower aminoglycoside MIC_50_ and MIC_90_ values (0.5 µg/mL and 1 µg/mL for gentamicin, 2 µg/mL and 4 µg/mL for streptomycin, and 1 µg/mL and 4 µg/mL for kanamycin) than that noted for isolates from livestock (0.5 µg/mL and 2 µg/mL for gentamicin, 4 µg/mL and 64 µg/mL for streptomycin, and 2 µg/mL and 8 µg/mL for kanamycin). With regard to resistance phenotype, only seven (8.1%) isolates were resistant to both streptomycin and kanamycin. In general, 55 (64%) *T. pyogenes* isolates were susceptible to all tested aminoglycosides.

### 2.2. Distribution of Integrons and Gene Cassettes in Studied T. pyogenes Isolates

All 86 *T. pyogenes* isolates were studied with regard to finding potential genetic determinants of aminoglycoside resistance. The class 1 *intI* gene associated with the class 1 integrons was detected in 30 (34.9%) *T. pyogenes* isolates. That confirmed a presence of those mobile elements in the genome of studied bacteria. However, the class 2 *intI* gene associated with the class 2 integrons was not found. Interestingly, the class 1 integrons were present in all isolates from small ruminants (goats and sheep) and in 43% of swine isolates, but only in two (7.7%) bovine isolates (Table S1). Importantly, the class 1 integrons were also found in six (24%) isolates from European bison; however, no gene cassettes were detected in those mobile elements (Table S1). The class 1 integron gene cassettes were present only in nine *T. pyogenes* isolates, including seven isolates from swine, one from cattle, and one from goat (Table S1). In all nine cases, PCR products specific for gene cassette, with a size approximately 850 bp, were sequenced. The analysis of the obtained sequences showed a presence of two aminoglycoside resistance genes, *aadA9* and *aadA11*, which were not previously reported in *T. pyogenes*. Thus, generally the class 1 integron gene cassettes associated with the aminoglycoside resistance were identified in 10.5% (9/86) of isolates.

### 2.3. Structure of the Class 1 Integron Gene Cassettes Associated with Aminoglycoside Resistance

**Figure 1:**
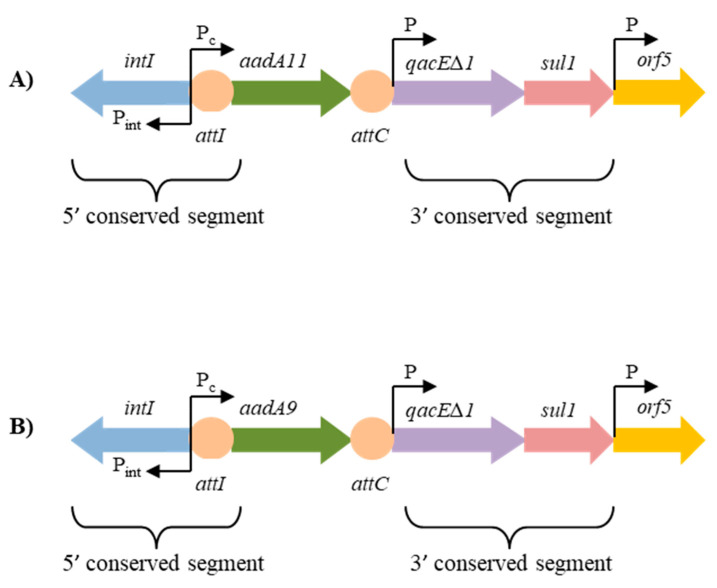
A schematic organization of the class 1 integrons and the aminoglycoside resistance gene cassettes *aadA11* (**A**) and *aadA9* (**B**) found in studied *T. pyogenes* isolates. Designations: *intI*, the integrase gene; *attI*, the recombination site of integrase; *aadA9* and *aadA11*, the streptomycin/spectinomycin resistance genes encoding adenylotransferases; *attC* the recombination site of cassette; *qacE∆1*, the quaternary ammonium compounds resistance gene; *sulI*, the sulfonamide resistance gene; *orf5*, a gene of unknown function; P, promoter; P_c_, cassette promoter; P_int_, integron promoter.

The sequence analysis of the class 1 integron gene cassettes found in *T. pyogenes* isolates revealed that they contained the *aadA9* (837 bp) or *aadA11* (846 bp) genes, encoding two adenyltransferases, AadA9 and AadA11, respectively, which may determine the streptomycin and spectinomycin resistance (Figure 1). Both those adenyltransferases belong to the ANT(3’’)-Ia family of aminoglycoside nucleotidyltransferases. The gene cassette containing the *aadA9* gene was present in one caprine and one bovine isolate, but the gene cassette with the *aadA11* gene was the most prevalent and occurred in seven swine isolates (Table S1). Importantly, this is the first description of the aminoglycoside adenyltransferase gene cassettes *aadA9* and *aadA11* in *T. pyogenes*. Moreover, the sequences of both genes, *aadA9* and *aadA11*, were deposited in the GenBank.

Each class 1 integron always contains *qacE∆1* (the quaternary ammonium compounds resistance gene), *sul1* (the sulfonamide resistance gene), and *orf5* (a gene of unknown function) in the 5’ conserved segment and the *intI* gene in the 3’ conserved segment [16]. In addition, the *attI* recombination site is located at the end of the 5’ conserved segment. Sequences of *attI* and *attC*, sites related to the gene cassette, may vary between different strains [28,29]. Figure 1 presents a schematic organization of the class 1 integron gene cassettes *aadA11* and *aadA9* detected in *T. pyogenes* in this study.

**Figure 2:**
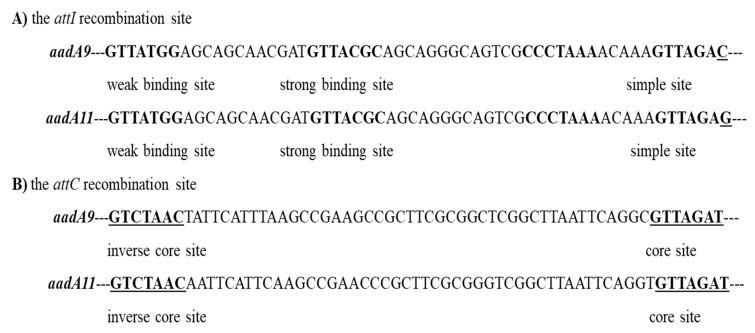
(**A**) Nucleotide sequences of the *attI* site associated with the class 1 integron gene cassettes *aadA9* and *aadA11* in studied *T. pyogenes* isolates. The weak, strong, and simple sites of the class 1 integrase binding are in bold. Differences in one nucleotide in the *attI* site sequences between *aadA9* and *aadA11* cassettes are in bold and underlined. (**B**) Nucleotide sequences of the *attC* sites associated with the class 1 integron gene cassettes *aadA9* and *aadA11* in *T. pyogenes*. Sequences of the inverse core sites and core sites for both gene cassettes are in bold and underlined.

In the investigated class 1 integron gene cassettes, both *attI* sites were 58 bp sequences, with the difference in one nucleotide, a cytosine in *addA9* and guanine in *aadA11* (Figure 2A). The *attC* site sequences, length 60 bp, were the same for both studied genes, and were composed of an inverse core site (GTCTAAC) and a core site (GTTAGAT) downstream of the genes (Figure 2B).

### 2.4. Prevalence of the Aminoglycoside Resistance Genes in Studied T. pyogenes Isolates

The prevalence of selected aminoglycoside resistance genes among studied *T. pyogenes* isolates was investigated by PCR. The results are presented in Table S1 and distribution of the tested resistance genes depending on an isolate origin is shown in Table 3. The *aadA9* and *aadA11* genes were present in eight (9.3%) and seven (8.1%) studied isolates, respectively (Figure S1 of Supplementary Materials). The *aadA11* gene was detected only in *T. pyogenes* swine isolates (7/21), and in all cases was carried on the class 1 integron gene cassettes. However, the *aadA9* gene only in two isolates, one bovine and one caprine, was located on the class 1 integron gene cassettes. In the remaining six isolates of bovine origin, *aadA9* was not associated with gene cassettes. The linked *strA*-*strB* genes were detected in a single bovine isolate (Figure S2 of Supplementary Materials), while the *aph(3’)-IIIa* gene was present in five isolates, one caprine and four swine (Figure S2). It should be highlighted that the *strA*-*strB* and *aph(3’)-IIIa* genes are reported in *T. pyogenes* for the first time. The remaining tested genes, *aadA1*, *aacC,* and *aac(6’)-aph(2’’)*, were not found in the studied isolates. Interestingly, any of the tested resistance genes were detected in the isolates from European bison, although two of them displayed the phenotypic resistance to kanamycin. Moreover, in the other 12 *T. pyogenes* isolates of various origin, also classified as phenotypically resistant to streptomycin or kanamycin, none of the tested resistance genes were detected.

## 3. Discussion

Aminoglycosides are antimicrobials commonly used to treat infections in various animals. Furthermore, gentamicin, spectinomycin, dihydrostreptomycin, and neomycin occur in veterinary formulations used in food-producing animals [30]. Nowadays, the treatment of bacterial infections becomes increasingly complicated due to the emergence and spread of resistant pathogens, including aminoglycoside-resistant *T. pyogenes* strains. Different mechanisms, often related to mobile genetic elements, contribute to dissemination of antimicrobial resistance. The significance of integrons and gene cassettes in spreading of drug resistance among bacteria has been a well-known fact. Class 1 integrons are one of the most important mobile genetic elements because they can capture and express diverse resistance genes in Gram-negative and Gram-positive bacteria [17,18].

*T. pyogenes* resistance to aminoglycosides was previously reported in limited publications. In this study, as shown in Table 1, all tested *T. pyogenes* isolates were susceptible to gentamicin, but 32.6% and 11.6% were resistant to streptomycin and kanamycin, respectively. In particular, a high frequency of resistant isolates was noted in cattle and swine. Similarly, in Iran, the majority of *T. pyogenes* bovine isolates was susceptible to gentamicin (98.5%) while 33.8% were streptomycin-resistant [31]. Pohl et al. [32] also observed a significant level of susceptibility to gentamicin (MIC_50_ = 1 µg/mL and MIC_90_ = 4 µg/mL) in *T. pyogenes* isolated from dairy cattle in Germany. On the other hand, a high percentage (84.4%) of streptomycin-resistant bovine *T. pyogenes* isolates was recorded in the study of Liu et al. in China [5]. Moreover, 40.6% and 18.8% of those isolates were resistant to amikacin and gentamicin, respectively [5]. Other studies also showed a higher level of resistance to gentamicin in *T. pyogenes* bovine isolates [33,34,35]. Interestingly, a significantly high percentage of strains resistant to aminoglycosides, such as gentamicin (77.8%), kanamycin (77.8%), amikacin (74.1%), streptomycin (77.8%), and spectinomycin (63%), was reported in case of *T. pyogenes* isolated from pigs in China [15,36]. The similar observation was reported by Galán-Relaño et al. for *T. pyogenes* swine isolates in Spain, characterized by high MICs of neomycin and streptomycin but low MICs of gentamicin and apramycin [37]. Moreover, Yoshimura et al. [4] reported that resistance to dihydrostreptomycin appeared more frequently among porcine isolates (85.7%) than among bovine isolates (52.4%). Resistance to gentamicin was noted only in 7.3% of bovine isolates [4]. On the other hand, all *T. pyogenes* swine isolates studied in Brazil were susceptible to gentamicin and streptomycin, whereas 13.3% of them were resistant to neomycin [38]. In contrast, the prevalence of aminoglycoside resistance in *T. pyogenes* strains isolated from wild animals seems to be significantly lower than in livestock isolates. Zhao et al. [7] noted a relatively low prevalence of resistance to streptomycin (21.4%), gentamicin (17.9%), amikacin (25.0%), apramycin (17.9%), and kanamycin (25%) among *T. pyogenes* strains isolated from forest musk deer in China. Similarly, *T. pyogenes* obtained from white-tailed deer in the United States were susceptible to gentamicin (86.2%) and spectinomycin (100%) [39]. Our results confirmed those observations as the MIC_50_ and MIC_90_ values of tested aminoglycosides determined for the isolates from European bison were clearly lower than that for the livestock isolates, and were 0.5 µg/mL and 1 µg/mL for gentamicin, 2 µg/mL and 4 µg/mL for streptomycin, and 1 µg/mL and 4 µg/mL for kanamycin, respectively.

An interpretation of antimicrobial susceptibility testing results in case of *T. pyogenes* is problematic, because currently there are no species-specific breakpoints established for this bacterium [40]. Moreover, limited data is available on MICs of different antimicrobials and on resistance mechanisms in that species. Generally, there is a lack of data that is essential for establishment of epidemiological cut-off values and clinical breakpoints for antimicrobials commonly used to treat *T. pyogenes* infections, including aminoglycosides. Thus, an appropriate classification of *T. pyogenes* isolates as susceptible or resistant is usually difficult. In this study, MIC breakpoints approved for *Corynebacterium* spp. and coryneforms [40] as well as the criteria previously used by Dong et al. [15] were applied to interpret susceptibility testing results for gentamicin, streptomycin, and kanamycin, respectively. Our study provided new important data on MIC value ranges of gentamicin, streptomycin, and kanamycin determined for *T. pyogenes* isolates from livestock and wild animals. The obtained results may be helpful for development of *T. pyogenes* specific breakpoints for aminoglycosides used in veterinary medicine. However, further investigations are required to approve new interpretive criteria for antimicrobial susceptibility testing in *T. pyogenes*.

In the present study, the screening for integrons in the genome of *T. pyogenes* isolates revealed that 30 (34.9%) of them carried the class 1 integrons. Importantly, in most cases the integron-positive isolates were from small ruminant and swine origin. Similarly, a high prevalence (approximately 60%) of the class 1 integron was noted in strains isolated from pigs [15] and forest musk deer [7] in China. In contrast, Liu et al. [5] reported a higher prevalence (50%) of the class 1 integrons among *T. pyogenes* bovine isolates also in China. In our study, the class 2 *intI* gene was not detected, indicating that the class 2 integrons were not present in tested *T. pyogenes* isolates. Those data are in agreement with previous reports from China [5,7,15], that also showed the lack of the class 2 integrons in the bacterium.

Interestingly, 17 out of 30 studied isolates containing the class 1 integrons were susceptible to all tested aminoglycosides. The sequence analysis of the integron variable region revealed that the class 1 integrons carried gene cassettes harboring aminoglycoside resistance genes only in nine out of 30 isolates. Thus, these findings indicated a high prevalence (70%) of the empty class 1 integrons among tested *T. pyogenes* isolates. Several other reports also showed a low frequency of gene cassettes’ occurrence in *T. pyogenes* isolates containing the class 1 integrons [5,7,15]. The class 1 integron gene cassettes may contain different aminoglycoside resistance genes, often depending on an origin of strains. In China, bovine *T. pyogenes* strains from endometritis, resistant to aminoglycosides, contained the *aadA1*, *aadA5*, *aadA24,* and *aadB* genes in the class 1 integron gene cassettes [5]. However, aminoglycoside resistance genes, such as *aadA1*, *aadA2*, *aadA4*, *aadA5*, *aac(6’)*-*Ib,* and *aac(6’)*-*IIc*, were found in the class 1 gene cassettes of swine isolates also from China [15]. Moreover, the *aadA1*, *aadA2,* and *aacC* genes associated with gene cassettes were also prevalent among *T. pyogenes* strains isolated from captive forest musk deer [7]. The same genes, *aadA1*, *aadA2,* and *aacC,* carried on gene cassettes were the most frequent among *T. pyogenes* mastitis and metritis bovine isolates in Iran [35]. All these findings suggest that *T. pyogenes* has a relatively high ability to acquire antimicrobial resistance genes carried by mobile elements such as gene cassettes. Our results confirm that suggestion, showing the presence of two aminoglycoside resistance genes, *aadA9* and *aadA11*, carried by the class 1 integron gene cassettes, which may occur in various bacteria but were not previously described in the species.

One of the aminoglycoside resistance mechanisms found in *T. pyogenes*, based on an antibiotic inactivation, is associated with the presence of *aadA* genes that encode ANT(3’’)-Ia family of aminoglycoside nucleotidyltransferases, determining resistance to streptomycin and spectinomycin. Interestingly, in our study we detected two novel aminoglycoside resistance determinants, *aadA9* and *aadA11*, belonging to the *aadA* gene group, that were not previously reported in *T. pyogenes*. Although it should be highlighted that *aadA11* was carried by the class 1 integron gene cassette in all cases, *aadA9* was associated with that mobile element only in 2/8 cases. Both those genes were present in the streptomycin-resistant *T. pyogenes* isolates from livestock animals. However, *aadA11* was prevalent in swine isolates, and *aadA9* was found in bovine and goat isolates. The nucleotide sequences of the *aadA9* genes found in *T. pyogenes* showed 100% identity to the *aadA9* gene in the integron structure of pTET3 from *Corynebacterium glutamicum* (GenBank No. NG_047368.1) [25]. The *aadA9* gene was also reported in other Gram-positive bacterium, *Arthrobacter arilaitensis*, isolated from agricultural soil in the United Kingdom (GenBank No. FJ457611.1) [41]. On the other hand, the *aadA11* gene detected in our *T. pyogenes* swine isolates presented 100% identity to the *aadA11* gene of the *Pseudomonas aeruginosa* human isolate from France (GenBank No. NG_047330.1) [42]. The *aadA11* gene was also identified in *P. aeruginosa* and *Alcaligenes faecalis* strains collected from swine environments on three Danish pig farms [43], as well as in *Escherichia coli* clinical strains isolated from humans in France [44]. Those data show a possibility of the *aadA9* and *aadA11* genes’ transmission between *T. pyogenes* and other Gram-positive bacteria as well as some Gram-negative species. However, further investigations must be performed to confirm the phenomenon.

A prevalence of the remaining resistance determinants tested in our research was significantly low. The *strA-strB* genes, encoding the streptomycin kinases APH(3’’)-Ib and APH(6’)-Id, respectively, were detected in a single bovine isolate, for which the streptomycin MIC value was 64 μg/mL. To the best of our knowledge, in this study the linked *strA-strB* genes were described in *T. pyogenes* for the first time. However, Dong et al. tested the presence of *strB* in *T. pyogenes*, but that gene was not detected in any of the isolates [15]. It is worth noting that the *strA* and *strB* genes are most often linked [45]. The primer set used in our study was designed to detect both those genes, since a primer forward is complementary to the final fragment of the *strA* gene. Thus, only the partial sequence of *strB* is a main PCR product. However, the presence of the *strA* gene in the studied isolate should be confirmed by sequencing of longer than a 33-nt fragment of that gene. The *strB* partial nucleotide sequence of *T. pyogenes*, reported in this study, showed 100% identity to the *strB* gene sequence of many bacterial species, such as *Salmonella enterica* subsp. *enterica* (Gen Bank No. CP019649.1), *E. coli* (GenBank No. JF916464.1), *Klebsiella pneumoniae* (GenBank No. JX442976.1), and other. Interestingly, the *strB* gene (also known as *aph(6)-Id*) is mainly distributed among Gram-negative bacteria [46,47,48,49,50,51]. In case of Gram-positive bacteria, that gene was only reported in human opportunistic pathogen *Corynebacterium striatum* [52] and *Streptococcus thermophilus* isolated from food [53]. The *strB* gene is usually carried by the transposon Tn*5393*, plasmids, or chromosomal genomic islands [49,50,51]. Additionally, in our study, the *aph(3’)-IIIa* gene, encoding the kinase APH(3’)-IIIa determining kanamycin resistance, was detected in four kanamycin-resistant isolates (MIC 8 µg/mL) and, surprisingly, in one isolate classified as kanamycin susceptible (MIC 4 µg/mL). Thus, that observation suggests that results of kanamycin susceptibility testing should be interpreted carefully and perhaps lower kanamycin MIC breakpoints for *T. pyogenes* should be considered. The *aph(3’)-IIIa* gene was also detected in *Lactobacillus bulgaricus* [53], *Enterococcus* spp., and *Staphylococcus aureus* [54]. To the best of our knowledge, the *aph(3’)-IIIa* gene was not previously reported in *T. pyogenes*.

In the current study, the relevance of the streptomycin and kanamycin resistance phenotype and genotype was not perfectly matched. In case of 14 (16.3%) *T. pyogenes* isolates demonstrating phenotypic resistance to streptomycin or kanamycin, we did not detect any of the tested aminoglycoside resistance genes, which could account for the phenotype. It is noteworthy that four of those resistant isolates and 17 other susceptible isolates harbored the empty class 1 integrons, which potentially may capture resistance gene cassettes. Such empty integrons were also found in other bacteria, for example in *E. coli* and *Enterococcus* spp. [55,56]. All those findings indicate a need of further investigations to assess other determinants and mechanisms of aminoglycoside resistance in *T. pyogenes*.

Concluding, the aminoglycoside resistance in *T. pyogenes* may be determined by the resistance mechanisms associated with production of aminoglycoside-modifying enzymes, such as ANTs and APHs, encoded by the genes *aad9*, *aad11,* and *strA-strB*, *aph(3’)-IIIa*, respectively. Those genes, described for the first time in *T. pyogenes* in our study, should be considered as significant genetic determinants involved in aminoglycoside resistance in the bacterium. The aforementioned genes were found mainly in *T. pyogenes* isolated from cattle and swine; therefore, both those animal species appear to be the most important reservoir of aminoglycoside-resistant strains. It seems especially interesting that some of the genes detected in *T. pyogenes* can be carried by the class 1 integron gene cassettes, indicating a high probability of their acquisition from other bacterial species, including Gram-negative bacteria. On the other hand, *T. pyogenes* has to be perceived as an important reservoir of aminoglycoside resistance genes. Moreover, it should be noted that our study provided the first data on the occurrence and prevalence of integrons and gene cassettes, as well as their sequences, among *T. pyogenes* strains isolated in Poland.

## 4. Materials and Methods

### 4.1. Bacterial Strains and Culture Conditions

A total of 86 *T. pyogenes* isolates from livestock and wild animals in Poland were studied (cattle = 26, pigs = 21, European bison = 25, goats = 8, sheep = 5, antelope = 1). Clinical specimens were collected from animals with different types of infection (mastitis, metritis, balanoposthitis, pneumonia, abscesses in various tissues). Isolates were cultured on Columbia Agar supplemented with 5% sheep blood (CA; Graso Biotech, Starogard Gdański, Poland) at 37 °C in 5% CO_2_ conditions for 48 h. All isolates were identified based on their phenotypic properties, as described in the previous studies [11,57].

Reference strains *T. pyogenes* ATCC^®^19411, *T. pyogenes* ATCC^®^49698, *E. coli* ATCC^®^25922, and *S. aureus* ATCC^®^25923 were used as quality controls of antimicrobial susceptibility testing.

### 4.2. Antimicrobial Susceptibility Testing

The MIC values of gentamicin (Sigma, Steinheim, Germany), streptomycin (Sigma, Steinheim, Germany), and kanamycin (Sigma, Steinheim, Germany) were determined using the standard broth microdilution method, according to the guidelines of the Clinical and Laboratory Standards Institute (CLSI) [40]. Briefly, the bacterial inoculum (approximately 4 × 10^5^ CFU/mL) was prepared in a sterile Müeller-Hinton broth (Difco, Franklin Lakes, NJ, USA) supplemented with 5% (*v*/*v*) fetal calf serum (Graso Biotech, Starogard Gdański, Poland), and 100 µL was added into 96 wells of microtiter plates. Serial double dilutions of tested antibiotics were performed in the same medium, then were added into the wells (100 µL/well) to receive a final antibiotic concentration over the range 0.125 to 128 µg/mL, respectively. Plates were incubated at 37 °C with 5% CO_2_ for 24 h. A MIC value was defined visually as the lowest antibiotic concentration in which bacterial growth was not observed. Currently, there are no aminoglycoside MIC breakpoints officially approved for *T. pyogenes*. Therefore, the results of MIC testing for gentamicin were interpreted according to the CLSI criteria approved for *Corynebacterium* spp. and coryneforms [40]. However, MIC breakpoints used previously by Dong et al. [15] for antimicrobial susceptibility testing in *T. pyogenes* were also applied in our study to interpret the MIC testing results for streptomycin and kanamycin.

### 4.3. Detection of Integrons and Gene Cassettes

A template DNA was extracted from studied *T. pyogenes* isolates cultured on CA after 48 h of incubation. For each isolate, colonies were suspended in 500 µL of distilled water and incubated at 99 °C for 10 min. Then, the sample was cooled on ice and centrifuged at 15,348 rcf (relative centrifugal force) for 6 min. A supernatant was collected and stored at −20 °C until further use.

The presence of the class 1 and class 2 integrons was confirmed by detection of the integron-encoded integrase genes, class 1 *intI* and class 2 *intI*, respectively. All isolates were tested by PCR technique using specific primers (Table 4). The presence of gene cassettes was also investigated by PCR using primer sets listed in Table 4. All PCR reaction mixtures with final volume of 25 µL contained: 8.5 µL of nuclease-free water (Thermo Fisher Scientific, Waltham, MA, USA), 12.5 µL of DreamTaq Green PCR Master Mix (Thermo Fisher Scientific, Waltham, MA, USA), 10 pmol (picomoles) of each primer (Genomed, Warsaw, Poland), and 2 µL (70–90 ng) of the DNA template. The thermal cycling conditions included initial denaturation at 95 °C for 3 min followed by 30 cycles of DNA denaturation at 95 °C for 30 sec, annealing for 30 sec at variable temperatures, as shown in Table 4, and extension at 72 °C for 1 min with a final extension step of 72 °C for 5 min. Amplification products were recognized by electrophoresis (85 V by 45 min) in 0.8% (*w*/*v*) agarose gel in Tris-Acetate-EDTA (TAE) buffer with Midori Green DNA Stain (Nippon Genetics, Düren, Germany), and visualized and analyzed using a VersaDoc Model 1000 Imaging System and Quantity One software (version 4.4.0) (Bio-Rad, Hercules, CA, USA). All amplicons of the class 1 integron gene cassettes were sequenced (Genomed, Warsaw, Poland) and a Basic Local Alignment Search Tool (BLAST) analysis was carried out on the National Center for Biotechnology Information (NCBI) web site (http://blast.ncbi.nlm.nih.gov) [58].

### 4.4. Detection of Aminoglycoside Resistance Genes

PCR was used to detect genes *aadA1*, *aadA9*, *aadA11*, *aacC*, *strA-strB*, *aph(3’)-IIIa,* and *aac(6’)-aph(2’’)*, encoding selected aminoglycoside resistance determinants. A composition of reaction mixtures was the same as described for amplification of integrons and gene cassettes, but with primers (Genomed, Warsaw, Poland) specific for studied genes (Table 4). Primers for the *aadA9* and *aadA11* genes were designed based on the obtained class 1 integron gene cassettes’ sequences. Primers for the *aadA1* and *aacC* genes were designed based on the known sequences of those genes deposited in GenBank, JN084184 and JN084183, respectively. The primers were designed using Primer-Blast (*https://www.ncbi.nlm.nih.gov/tools/primer-blast/*). PCR thermal cycling conditions were as follows: An initial denaturation at 95 °C for 3 min followed by 35 cycles of DNA denaturation at 95 °C for 30 sec, annealing for 30 sec at different temperatures, as shown in Table 4, and extension at 72 °C for 45 sec with a final extension step of 72 °C for 2 min (for *aadA1*, *aadA9*, *aadA11*, *aacC*, *aac(6’)-aph(2’’)*) and 5 min (for *strA*-*strB* and *aph(3’)-IIIa*). Products of the amplification were separated by electrophoresis (85 V by 45 min) in 0.8% (*w*/*v*) agarose gel in TAE buffer with Midori Green DNA Stain (Nippon Genetics, Düren, Germany), and visualized and analyzed using a VersaDoc Model 1000 Imaging System and Quantity One software (version 4.4.0) (Bio-Rad, Hercules, CA, USA). The clinical isolates, *K. pneumoniae* 15/BM, *P. aeruginosa* 420/BM, *Pediococcus pentosaceus* WN1 and *E. coli* pMW10, were used as positive controls in the PCR reactions for *aadA1*, *aacC*, *strA*-*strB,* and *aph(3’)-IIIa*, respectively.

A single primer set was used to detect the linked *strA*-*strB* genes in PCR. The amplicon obtained in that reaction, corresponding mainly to a first part of the *strB* gene, was sequenced (Genomed, Warsaw, Poland) and a Basic Local Alignment Search Tool (BLAST) analysis was carried out on the National Center for Biotechnology Information (NCBI) web site (http://blast.ncbi.nlm.nih.gov) [58].

### 4.5. Nucleotide Sequence Accession Numbers

The *aadA9* and *aadA11* gene sequences, as well as the partial sequence of the *strB* gene obtained in the study were deposited in GenBank (https://www.ncbi.nlm.nih.gov/genbank) under accession numbers MN171325, MN171326, and MT500573, respectively.

## Figures and Tables

**Table 1:** Distribution of minimum inhibitory concentration (MIC), MIC_50,_ and MIC_90_ values of three aminoglycoside antibiotics among studied *Trueperella pyogenes* isolates (*n* = 86).

Antibiotic	Number of Isolates with MIC (µg/mL)^*^											MIC_50_	MIC_90_
	≤0.125	0.25	0.5	1	2	4	8	16	32	64	≥128		
Gentamicin	9	12	35	18	12			│				0.5	2
Streptomycin					41	17│	8	4	4	9	3	4	64
Kanamycin	2	6	12	23	20	13│	5	2	3			1	8

^*^MIC breakpoints used in this study (vertical bars): Gentamicin ≥ 16 µg/mL, streptomycin > 4 µg/mL, kanamycin > 4 µg/mL; numbers of resistant isolates are in bold.

**Table 2:** Prevalence of the aminoglycoside resistance in studied *Trueperella pyogenes* isolates (*n* = 86).

Antibiotic	Total (*n* = 86)	Resistant Isolates From [% (*n*)]					
		Cattle (*n* = 26)	Pigs (*n* = 21)	European bison (*n* = 25)	Goats (*n* = 8)	Sheep (*n* = 5)	Antelope (*n* = 1)
Gentamicin	0 (0)	0 (0)	0 (0)	0 (0)	0 (0)	0 (0)	0 (0)
Streptomycin	32.6 (28)	53.8 (14)	57.1 (12)	0 (0)	12.5 (1)	20.0 (1)	0 (0)
Kanamycin	11.6 (10)	11.5 (3)	23.8 (5)	8,0 (2)	0 (0)	0 (0)	0 (0)

**Table 3:** Distribution of the aminoglycoside resistance genes in studied *Trueperella pyogenes* isolates (*n* = 86).

Resistance Gene	Total (*n* = 86)	Isolates From [% (*n*)]					
		Cattle (*n* = 26)	Pigs (*n* = 21)	European Bison (*n* = 25)	Goats (*n* = 8)	Sheep (*n* = 5)	Antelope (*n* = 1)
*aadA1*	0 (0)	0 (0)	0 (0)	0 (0)	0 (0)	0 (0)	0 (0)
*aadA9*	9.3 (8)	27.0 (7)	0 (0)	0 (0)	12.5 (1)	0 (0)	0 (0)
*aadA11*	8.1 (7)	0 (0)	33.3 (7)	0 (0)	0 (0)	0 (0)	0 (0)
*aacC*	0 (0)	0 (0)	0 (0)	0 (0)	0 (0)	0 (0)	0 (0)
*strA-strB*	1.2 (1)	3.8 (1)	0 (0)	0 (0)	0 (0)	0 (0)	0 (0)
*aph(3’)-IIIa*	5.8 (5)	0 (0)	19.0 (4)	0 (0)	12.5 (1)	0 (0)	0 (0)
*aac(6’)-aph(2’’)*	0 (0)	0 (0)	0 (0)	0 (0)	0 (0)	0 (0)	0 (0)

**Table 4:** Primers used in this study.

Amplification Target	Primers Sequence (5’-3’)	Annealing Temperature (°C)	PCR Product size (bp)	Reference
*Class 1 intI*	F: CCTCCCGCACGATGATC	57	280	[7]
	R: TCCACGCATCGTCAGGC			
*Class 2 intI*	F: TTATTGCTGGGATTAGGC	50	233	[7]
	R: ACGGCTACCCTCTGTTATC			
*Class 1 gene cassette*	F: GGCATCCAAGCAGCAAG	58	unpredictable	[15]
	R: AAGCAGACTTGACCTGA			
*aadA1*	F: CGGTGACCGTAAGGCTTGAT	52	193	This study
	R: ATGTCATTGCGCTGCCATTC			
*aadA9*	F: ACGCCGACCTTGCAATTCT	52	373	This study
	R: TAGCCAATGAACGCCGAAGT			
*aadA11*	F: CGTGCATTTGTACGGCTCTG	53	352	This study
	R: ACCTGCCAATGCAAGGCTAT			
*aacC*	F: TTGCTGCCTTCGACCAAGAA	53	256	This study
	R: TCCCGTATGCCCAACTTTGT			
*strA-strB*	F: TATCTGCGATTGGACCCTCTG	60	538	[46]
	R: CATTGCTCATCATTTGATCGGCT			
*aph(3’)-IIIa*	F: GGCTAAAATGAGAATATCACCGG	55	523	[59]
	R: CTTTAAAAAATCATACAGCTCGCG			
*aac(6’)-aph(2’’)*	F: CCAAGAGCAATAAGGGCATA	48	220	[60]
	R: CACTATCATAACCACTACCG			

## Supplementary Materials

Supplementary materials can be found at https://www.mdpi.com/1422-0067/21/12/4230/s1.
