# Supplementary material for: New Determinants of Aminoglycoside Resistance and Their Association with the Class 1 Integron Gene Cassettes in Trueperella pyogenes
Source: Int J Mol Sci. 2020 Jun 13;21(12):4230. doi: 10.3390/ijms21124230 (PMC7352783; doi:10.3390/ijms21124230)
Supplement: Supplementary file 1 [file ijms-21-04230-s001.zip › Supplementary Materials - Rzewuska Magdalena/Figure S1.pdf]

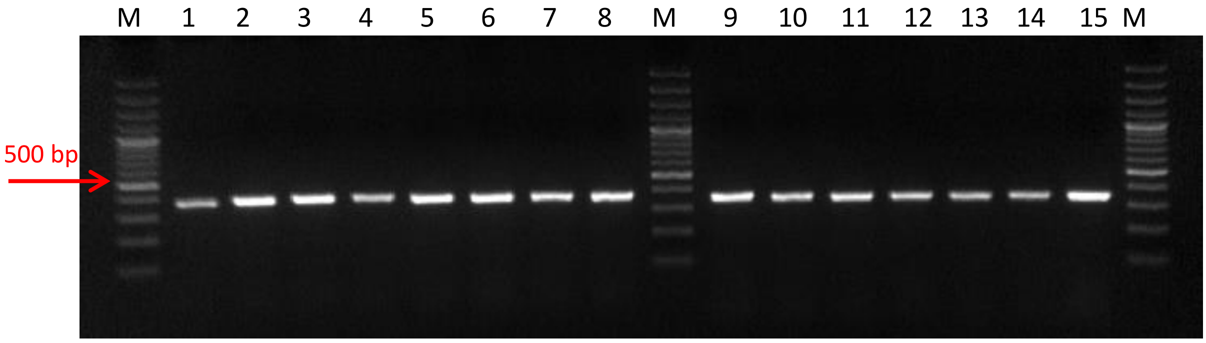

Figure S1. Results of electrophoresis after PCR detection of the *aadA9* and *aadA11* genes. M: GeneRuler 100 bp Plus DNA Ladder (Thermo Fisher Scientific, Waltham, Massachusetts, USA); lines 1–8: the *aadA9* amplicons (6/K, 2/B, 3/B, 4/B, 7/B, 21/B, 23/B, 25/B *T. pyogenes* isolates, respectively), lines 9–15: the *aadA11* amplicons (4/S, 7/S, 8/S, 9/S, 15/S, 17/S, 18/S *T. pyogenes* isolates, respectively).
