# Supplementary material for: New Determinants of Aminoglycoside Resistance and Their Association with the Class 1 Integron Gene Cassettes in Trueperella pyogenes
Source: Int J Mol Sci. 2020 Jun 13;21(12):4230. doi: 10.3390/ijms21124230 (PMC7352783; doi:10.3390/ijms21124230)
Supplement: Supplementary file 1 [file ijms-21-04230-s001.zip › Supplementary Materials - Rzewuska Magdalena/Figure S2.pdf]

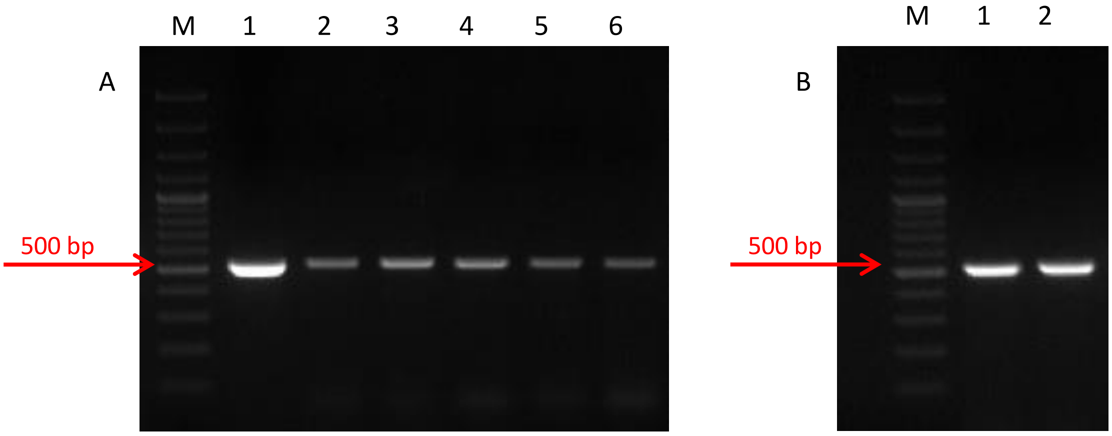

Figure S2. Results of electrophoresis after PCR detection of: (A) the *aph(3')-IIIa* gene; M: GeneRuler 100 bp Plus DNA Ladder (Thermo Fisher Scientific, Waltham, Massachusetts, USA); line 1: a positive control (*Escherichia coli* pMW10); lines 2–6: the *aph(3')-IIIa* amplicons (1/K, 9/S, 15/S, 17/S, 18/S *T. pyogenes* isolates, respectively); (B) the *strA-strB* genes; M: GeneRuler 100 bp Plus DNA Ladder (Thermo Fisher Scientific, Waltham, Massachusetts, USA); line 1: a positive control (*Pediococcus pentosaceus* WN1); line 2: The *strA-strB* amplicon of *T. pyogenes* isolate 9/B.
