# Supplementary material for: New Determinants of Aminoglycoside Resistance and Their Association with the Class 1 Integron Gene Cassettes in Trueperella pyogenes
Source: Int J Mol Sci. 2020 Jun 13;21(12):4230. doi: 10.3390/ijms21124230 (PMC7352783; doi:10.3390/ijms21124230)
Supplement: Supplementary file 1 [file ijms-21-04230-s001.zip › Supplementary Materials - Rzewuska Magdalena/Table S1.pdf]

Table S1. MIC values of tested antimicrobials and aminoglycoside resistance phenotypes and genotypes of studied *Trueperella pyogenes* isolates (n = 86).

Isolate*	MIC of gentamicin (μg/mL)	MIC of streptomycin (μg/mL)	MIC of kanamycin (μg/mL)	Resistance phenotype**	Resistance genotype
1/ANT	0.5	2	1	-	-
1/O	0.25	4	0.5	-	class 1 *intI*
2/O	0.25	4	4	-	class 1 *intI*
3/O	0.5	8	1	STR	class 1 *intI*
4/O	0.5	2	1	-	class 1 *intI*
5/O	0.5	2	2	-	class 1 *intI*
1/K	0.5	2	4	-	class 1 *intI*<br>*aph(3')-IIIa*
2/K	0.5	2	1	-	class 1 *intI*
4/K	0.5	2	2	-	class 1 *intI*
5/K	1	2	0.25	-	class 1 *intI*
6/K	1	>128	0.5	STR	class 1 *intI* – gene cassette – *aadA9*
7/K	0.25	4	0.5	-	class 1 *intI*
8/K	1	4	0.5	-	class 1 *intI*
9/K	1	4	1	-	class 1 *intI*
1/Z	0.5	2	1	-	-
2/Z	0.5	2	1	-	-
3/Z	0.5	2	4	-	-
4/Z	0.5	2	4	-	-
5/Z	0.5	2	1	-	-
6/Z	0.5	2	0.25	-	-
7/Z	0.5	4	≤0.125	-	-
8/Z	0.5	4	0.5	-	-
9/Z	0.25	2	1	-	-
10/Z	≤0.125	2	4	-	-
11/Z	0.25	2	32	KAN	class 1 *intI*
12/Z	0.25	2	2	-	-
14/Z	0.5	2	0.5	-	-
15/Z	0.5	2	2	-	-
16/Z	1	2	0.5	-	-
17/Z	1	2	≤0.125	-	-
18/Z	0.5	2	1	-	class 1 *intI*
19/Z	0.5	2	0.25	-	-
20/Z	0.5	2	4	-	class 1 *intI*
21/Z	0.5	4	2	-	class 1 *intI*
22/Z	0.5	2	8	KAN	-
23/Z	0.5	4	2	-	-
24/Z	0.5	4	1	-	-
25/Z	0.5	4	2	-	class 1 *intI*
26/Z	1	4	4	-	class 1 *intI*
4/S	1	32	4	STR	class 1 *intI* – gene cassette – *aadA11*
5/S	1	4	2	-	-
6/S	1	2	1	-	class 1 *intI*

7/S	1	16	4	STR	class 1 *intI* – gene cassette – *aadA11*
8/S	1	16	16	STR – KAN	class 1 *intI* – gene cassette – *aadA11*
9/S	2	8	8	STR – KAN	class 1 *intI* – gene cassette – *aadA11* *aph(3')-IIIa*
10/S	2	16	1	STR	-
13/S	1	>128	0.5	STR	class 1 *intI*
14/S	1	64	0.5	STR	-
15/S	2	>128	8	STR – KAN	class 1 *intI* – gene cassette – *aadA11* *aph(3')-IIIa*
16/S	2	8	1	STR	-
17/S	2	8	8	STR – KAN	class 1 *intI* – gene cassette – *aadA11* *aph(3')-IIIa*
18/S	2	16	8	STR – KAN	class 1 *intI* – gene cassette – *aadA11* *aph(3')-IIIa*
19/S	1	64	1	STR	-
20/S	2	2	1	-	-
21/S	2	2	0.5	-	-
22/S	1	2	1	-	-
23/S	2	2	1	-	-
24/S	2	2	2	-	-
25/S	2	2	1	-	-
26/S	2	2	0.5	-	-
2/B	0.5	64	2	STR	*aadA9*
3/B	0.5	8	1	STR	*aadA9*
4/B	≤0.125	64	32	STR – KAN	*aadA9*
5/B	0.25	2	0.25	-	-
6/B	0.25	8	0.25	STR	-
7/B	0.25	8	2	STR	class 1 *intI* – gene cassette – *aadA9*
9/B	≤0.125	64	32	STR – KAN	*strA-strB*
11/B	≤0.125	2	2	-	-
12/B	0.25	4	1	-	-
13/B	0.25	8	4	STR	class 1 *intI*
14/B	0.5	4	0.25	-	-
15/B	≤0.125	4	2	-	-
16/B	≤0.125	2	2	-	-
18/B	0.25	4	2	-	-
19/B	≤0.125	2	1	-	-
20/B	≤0.125	2	2	-	-
21/B	≤0.125	32	2	STR	*aadA9*
22/B	0.5	32	1	STR	-
23/B	0.5	64	2	STR	*aadA9*
24/B	0.5	32	4	STR	-
25/B	0.5	64	0.5	STR	*aadA9*
26/B	1	64	2	STR	-
27/B	0.5	2	2	-	-
29/B	0.5	2	16	KAN	-
30/B	0.5	2	4	-	-
31/B	1	64	4	STR	-

T. pyogenes ATCC®19411	2	2	1	-	-
T. pyogenes ATCC®49698	1	4	1	-	-

* An isolate origin: ANT – antelope, O – sheep, K – caprine, Z – European bison, S – swine, B – bovine.
** Antimicrobial: STR – streptomycin, KAN – kanamycin.
